# Serum Concentrations of Cholinesterase Inhibitors in Patients With Alzheimer’s Dementia Are Frequently Below the Recommended Levels

**DOI:** 10.3389/fphar.2020.00691

**Published:** 2020-05-21

**Authors:** Marion Ortner, Marion Stange, Heike Schneider, Charlotte Schroeder, Katharina Buerger, Claudia Müller, Bianca Dorn, Oliver Goldhardt, Janine Diehl-Schmid, Hans Förstl, Werner Steimer, Timo Grimmer

**Affiliations:** ^1^Department of Psychiatry and Psychotherapy, School of Medicine, Klinikum rechts der Isar, Technical University of Munich, Munich, Germany; ^2^Institute for Clinical Chemistry and Pathobiochemistry, School of Medicine, Klinikum rechts der Isar, Technical University of Munich, Munich, Germany; ^3^Institut for Stroke and Dementia Research, University of Munich, Munich, Germany; ^4^DZNE – German Center for Neurodegenerative Diseases, Munich, Germany

**Keywords:** Alzheimer's disease, Alzheimer's dementia, cholinesterase inhibitors, serum concentration, therapeutic drug monitoring, treatment efficacy, gene dose, CYP2D6 polymorphism

## Abstract

**Background:**

Acetylcholinesterase inhibitors (AChE-I) are recommended for the treatment of cognitive symptoms but also of behavioral and psychological symptoms in dementia. They are widely used not only in Alzheimer's disease, but also in other forms of dementia. Efficacy of treatment might depend on serum concentration of the respective AChE-I.

**Objective:**

In patients with mild to moderate Alzheimer's dementia, we measured serum concentrations of hepatically metabolized donepezil and renally excreted rivastigmine and investigated possible modifiers. Additionally, we looked at correlations between serum concentrations and efficacy for both drugs.

**Methods:**

Serum concentrations of donepezil and rivastigmine were measured by liquid chromatography – tandem mass spectrometry (LC-MS/MS). Real-time quantitative polymerase chain reaction (PCR). Allele specific PCR were performed to determine CYP2D6 genotype and gene dose. Clinical efficacy was assessed by changes of the subtest wordlist delayed recall of the Consortium to Establish a Registry for Alzheimer's Disease-Neuropsychological Assessment Battery (CERAD-NAB).

**Results:**

Sixty-seven patients treated with a stable dosage of donepezil 10 mg (n=41) or rivastigmine 9.5 mg (n=26) were included. Mean serum concentration of donepezil and rivastigmine were 41.2 and 6.5 ng/ml, respectively. Serum concentrations were below the recommended range in 73% of the subjects in the donepezil group and in 65% of the participants in the rivastigmine group. When applying a dose-related reference, ranges 63% of patients in the donepezil group and 32% in the rivastigmine group had concentrations below the expected range. Gene dose, sex, and duration of treatment significantly predicted donepezil serum concentration (p=0.046, p=0.001, p=0.030 respectively). Only for rivastigmine did the serum concentration significantly contribute to the regression model predicting changes on the subtest word list delayed recall (β=0.472; p=0.019).

**Conclusions:**

Serum concentrations of about two thirds of the patients were below the recommended range. When not looking at absolute values but at the dose-related reference ranges, these numbers improved but still 32%, respectively 63% of patients had low serum concentrations. High serum concentrations of rivastigmine predicted clinical response to cognition. Therapeutic drug monitoring might help to identify the cause of poor clinical response to cognition and behavioral and psychological symptoms in patients with AChE-I treatment.

## Introduction

Over 75% of dementia patients are affected by behavioral and psychological symptoms in dementia (BPSD) over the course of the disease. BPSD constitute a significant burden to caregivers and nursing staff (Lyketsos et al., 2002; Steinberg et al., 2008; Torrisi et al., 2017; Hessler et al., 2018). Symptoms like depression and anxiety are even associated with an increased suicide risk (Seyfried et al., 2011). For mild to moderate dementia due to Alzheimer's disease (ADD), the most common cause of dementia (van der Flier and Scheltens, 2005), symptomatic treatment with acetylcholinesterase inhibitors (AChE-I) such as donepezil, galantamine, or rivastigmine is recommended (Doody et al., 2001; Dimentia, 2007; Gauthier et al., 2012; Deuschl, 2016; Dyer et al., 2016). Rivastigmine additionally pseudo-irreversibly inhibits butyrylcholinesterase (BChE) (Weinstock, 1999), an enzyme that, like AChE, catalyzes the hydrolysis of acetylcholine. Besides the effect of AChE-I on cognitive symptoms, they can also improve BPSD not only in ADD (Cummings et al., 1994; Birks, 2006; Tan et al., 2014; Matsuzono et al., 2015; Kratz, 2017), but also in dementia in idiopatic Parkinson's disease (PDD) and dementia with Lewy bodies (DLB) (Rolinski et al., 2012; Mori et al., 2016). However, no improvement of BPSD has been detected under treatment with rivastigmine in one study (Birks et al., 2015). There is no evidence for the efficacy of AChE-I in frontotemporal lobar degeneration, multiple sclerosis, or supranuclear palsy (Li et al., 2015). As the efficacy of AChE-I probably depends on drug serum or plasma concentration (Rogers et al., 1998; Chou et al., 2012; Yang et al., 2013; Hefner et al., 2015; Manabe et al., 2016; Chen et al., 2017; Miranda et al., 2017), inconclusive results might be due to insufficient drug blood concentrations in some subjects.

Factors affecting plasma concentrations are sex (Noetzli et al., 2014), age (Coin et al., 2016; Mori et al., 2016), weight (FDA, 2012; FDA, 2015), dosage (Darreh-Shori et al., 2006; Mori et al., 2016), and duration of treatment (Miranda et al., 2017). The metabolism of the respective AChE-I, particularly for donepezil, might also have an impact on serum concentration. While rivastigmine is mainly hydrolyzed and renally excreted (FDA, 2012), donepezil is hepatically metabolized mainly by the cytochrome P450 isoenzyme CYP2D6 and, to a lesser extent, by CYP3A4 and undergoes glucuronidation (FDA, 2015). Treatment with CYP2D6 inhibitors or CYP2D6 inducers (FDA, 2015) as well as CYP2D6 polymorphisms can result in altered enzyme activity and thus altered metabolism rates (Meyer and Zanger, 1997; Sachse et al., 1997; Raimundo et al., 2004; Hicks et al., 2013). Depending on the genetically determined enzyme activity, patients can be classified by their phenotype as poor metabolizer (PM) with no enzyme activity, intermediate metabolizer (IM) with reduced enzyme activity, extensive metabolizer (EM) with normal enzyme activity, and ultra-rapid metabolizer (UM) with increased enzyme activity. Instead of correlating the phenotype with the genotype, Steimer et al. recommend to correlate the phenotype with a semiquantitative gene dose that depends on the number and the activity of detected CYP2D6 alleles (Steimer et al., 2004).

Which AChE-I is prescribed at what dose depends on local drug approval and patients' preferences, e. g. if an oral application or a transdermal patch is preferred, but mainly it is in the physician's responsibility to choose the best drug at the right dosage for the individual patient. To support this decision, guidelines provide information about recommended therapeutic reference ranges for blood concentrations (Hiemke et al., 2018).

In this study, we measured serum concentrations of the hepatically metabolized donepezil and the renally metabolized rivastigmine, looked at potential factors influencing serum concentrations such as CYP2D6 polymorphisms and investigated correlations between serum concentrations and clinical efficacy for both drugs.

## Materials and Methods

### Ethics Statement

The study protocol was approved by the ethics committee of the Faculty of Medicine of the Technical University of Munich, Munich (reference number 673/02). All patients provided written informed consent prior to any study specific procedures. All clinical investigations have been conducted in accordance with the principles of the Declaration of Helsinki, sixth revision.

### Patient Recruitment and Study Design

The study was conducted at the outpatient unit of the Centre for Cognitive Disorders at the Department of Psychiatry, Klinikum rechts der Isar, Technical University of Munich, Munich, Germany and at the Memory Clinic at the Institute for Stroke and Dementia Research, Klinikum der Universitaet Muenchen, Munich, Germany between October 2012 and December 2014. Patients had initially been referred for the diagnostic evaluation of cognitive impairments by self-referral, general practitioners, neurologists, psychiatrists, or other institutions, and had undergone a standardized diagnostic procedure that has been described previously (Ortner et al., 2015). It included an interview with the patient and an informant, obtaining demographic data, medical history, concomitant medication, physical, neurological, and psychiatric examinations, a neuropsychological evaluation including the Mini-Mental State Examination (MMSE) (Folstein et al., 1975), the Consortium to Establish a Registry for Alzheimer's Disease Neuropsychological Assessment Battery (CERAD-NAB) (Morris et al., 1989), as well as a routine laboratory screening test.

The treating physician started patients diagnosed with mild to moderate Alzheimer's dementia (McKhann et al., 2011) on treatment with an AChE-I by his own choice in accordance with the German treatment guidelines for Alzheimer's dementia (Deuschl, 2016). Mild dementia was defined by a MMSE of 26–20 points, moderate dementia by 19–10 points. German treatment guidelines for Alzheimer's disease recommend AChE-I such as donepezil, rivastigmine, or galantamine for symptomatic treatment of mild and moderate Alzheimer's dementia (Deuschl, 2016). Memantine is recommended for moderate and severe Alzheimer's dementia. Each doctor is independent in his or her choice which of the approved drugs is prescribed. At a routine follow-up appointment after 3–6 months, patients were informed about the study and asked to provide written informed consent if they met the inclusion criteria.

Patients needed to be able to provide written informed consent, actually have started treatment and be on stable medication for at least five half-times with either donepezil capsules 10 mg or rivastigmine transdermal patch 9.5 mg per day. They should be compliant in taking their medication as ascertained by caregiver information. The only study specific procedure consisted in a venous blood draw in order to assess serum concentrations of donepezil and rivastigmine and the genotype and gene dose of CYP2D6 alleles for subjects treated with donepezil. Routine follow-up procedures included a patient and caregiver interview with an assessment of the current medication, the start of AChE-I treatment, the time point when the dose of AChE-I was increased, side effects of AChE-I treatment, a neuropsychological assessment (CERAD-NAB, MMSE), and a psychiatric and neurological examination.

Patients were excluded from study participation if they were incapable to provide written informed consent, were not started or not on a stable dosage of donepezil or rivastigmine, or if dementia was due to any other disease than Alzheimer's disease, for instance Parkinson's disease. Further exclusion criteria were other possible causes of cognitive impairment such as sedating psychotropic medication (e.g. tricyclic antidepressants, low-potent antipsychotics), substance misuse, clinical signs of major depression, or major abnormalities in the routine blood testing at the initial presentation of the patient.

### Blood Sample Collection and Analyses

To measure steady-state trough drug levels patients needed to be on the respective AChE-I for at least five half-times and blood was drawn as close as possible to the time the next dose would have been due. If the blood sample could not be drawn prior to the next scheduled time point for dosing, medication was held until after the blood draw.

Serum concentrations of donepezil and rivastigmine were measured by liquid chromatography – tandem mass spectrometry (LC-MS/MS). Standard procedures were used for genotyping. Depending on allele status subjects were classified into PM, IM, EM, and UM (**Supplementary Table 1**). Additionally, as proposed by Steimer et al. (2004), to each allele an individual gene dose was assigned to calculate a semiquantitative gene dose (**Supplementary Tables 1–3**). Details of blood sample collection, LC-MS/MS method and genotyping and gene dose assignment are provided in **Supplementary Materials**.

In addition to the absolute serum concentrations, we also looked at the dose-related reference ranges for both treatment groups as defined by the Consensus Guidelines for Therapeutic Drug Monitoring in Neuropsychopharmacology (Hiemke et al., 2018). The dose-related reference range gives information about serum concentrations that can be expected for 68% of patients aged 18–65 years with a body weight of 70 kg at a given dose.

### Cognitive Assessments and Evaluation of Treatment Efficacy

Efficacy of the treatment with donepezil or rivastigmine was evaluated by changes of the CERAD-NAB sub-test wordlist delayed recall before initiation of an AChE-I and under stable treatment. The absolute difference in points scored in the initial and follow-up assessment was calculated. Negative values indicated worsening, positive values indicated improvement.

### Statistical Analyses

Subjects under treatment with donepezil 10 mg or rivastigmine 9.5 mg, respectively, were characterized using descriptive statistics. All statistics were calculated using SPSS Statistics 23 (SPSS Inc., Chicago, Il, USA). Group comparisons for normally distributed variables were calculated using t-test, for not normally distributed variables, Mann-Whitney U test was applied. A significance threshold of p < 0.05 (two sided) was applied.

In the donepezil group only, we assessed the correlation between serum concentration of donepezil and gene dose of CYP2D6 using multivariate linear regression analysis. The dependent variable was donepezil serum concentration; independent variables were chosen based on their known influence on donepezil concentration and consisted of gene dose, concomitant medication with CYP2D6 inhibitors, sex, age, weight, duration of treatment with donepezil, and time since last dosing. As a confirmative analysis, the same model was calculated using metabolism type instead of gene dose.

To investigate correlations between serum concentration and efficacy of the respective treatment, linear regression analyses with the difference between baseline to follow-up in the sub-test word list delayed recall as the dependent variable were calculated. Independent variables were serum concentration, results of delayed recall at the initial assessment, time between initial assessment and follow-up, sex, and age. To further investigate correlations between serum concentration and clinical efficacy, we repeated the regression analyses using low/high serum concentration of the respective AChE-I as a factor. The “low concentration” group consisted of the third of subjects with the lowest serum concentrations and the “high concentration” group of the third of subjects with the highest serum concentrations for the respective AChE-I.

We chose the sub-test word list delayed recall as endpoint for treatment efficiency. The rationale behind that decision was that (i) most variability could be expected in this test and it discriminates best between healthy controls and even mildly demented subjects (Welsh et al., 1991) and (ii) by focusing on a single endpoint, we wanted to avoid multiple comparisons. All statistical models were tested for interaction. There was no imputation of missing data.

## Results

### Characteristics of Participants

Characteristics of participants are shown in **Table 1**. Forty-one subjects were included in the donepezil group and 26 in the rivastigmine group. There were no statistically significant differences between the groups regarding sex, age, time between initial and follow-up assessment, duration of treatment with an AChE-I, weight, BMI (**Table 1**), or results of the neuropsychological assessments at baseline and follow-up (**Supplementary Table 4**). The progress of participants is shown in **Supplementary Figure 1**.

**Table 1:** Characteristics of participants: where applicable: mean ± standard deviation (minimum - maximum).

	Donepezil	Rivastigmine	p-value
Numbers	41	26	–
Sex: male:female	21: 20 (51%: 49%)	16:10 (61.5%: 38.5%)	0.458^a^
Age [years]	72.7 ± 9.51 (53-88)	72.1 ± 7.0 (53-86)	0.772^b^
Time baseline to follow up [days]	217.7 ± 64.8 (96-441)	228.7 ± 80.2 (122-442)	0.990^c^
Duration from start of AChE-I treatment to follow up [months]	5.7 ± 2.4 (3-13)	5.7 ± 2.1 (2-12)	0.825^c^
Number of participants taking CYP2D6 inhibitors	6 (5 x Citalopram; 1 x Carvedilol)	n.a.	–
Number of participants taking CYP2D6 inducers	none	n.a.	–
Weight [kg] n=37 (Donepezil) n=24 (Rivastigmine)	69.5 ± 8.5 (50-83)	68.2 ± 13.8 (47-90)	0.671^b^
BMI [kg/m²] n=37 (Donepezil) n=24 (Rivastigmine)	23.8 ± 3.0 (17.2-34.5)	23.5 ± 3.6 (16.5-30.9)	0.743^b^

AChE-I, acetylcholinesterase inhibitor; n.a., not applicable; kg, participants' weight in kilogram; BMI, body mass index; p-values calculated from ^a^Chi-squared test, ^b^t-test or ^c^Mann-Whitney-U-test.

### Drug Serum Concentrations and Gene Dose

#### Serum Concentrations of Donepezil and Rivastigmine

The next AChE-I administration was held until after the blood draw. On average, blood was drawn 1.32 ± 2.4 (-10 to +4) hours before the next scheduled drug administration. Drug serum concentrations are shown in **Table 2**. The variance of serum concentration of donepezil differed significantly from that of rivastigmine (p < 0.001) while variation coefficients did not significantly differ (p=0.775; **Table 2**).

**Table 2:** Serum concentrations of AChE-I: Daily drug dose, serum concentration, and variance of blood serum concentration for donepezil and rivastigmine group, respectively.

Variables	Donepezil 10 mg/d	Rivastigmine 9.5 mg/d	p-value
Serum concentration [ng/ml]	41.22 ± 15.56 (18.8-87.6)	6.53 ± 5.14 (0.47-17.50)	
Variance of serum concentration	242.0	26.5	<0.001^a^
Variation coefficient of serum concentration measurement	5.11 ± 2.58 (0.57-10.32)	5.57 ± 3.61 (1.00-17.40)	0.990^b^
Time since last dosing [h]	22.0 ± 2.2 (14-26)	23.6 ± 3.0 (15-28)	0.002^b^

h, hours; ng, nanogram; ml, milliliter; mg, milligram; d, day; where applicable: mean ± standard deviation (minimum – maximum). ^a^calculated from F-test. ^b^calculated from Mann-Whitney U test.

Female subjects had significantly higher donepezil serum concentrations (p=0.004) and lower absolute body weight (p < 0.001), while BMI did not statistical significantly differ between sexes (**Table 3**). In the rivastigmine group, serum concentrations did not differ between sexes (**Supplementary Table 5**). Female subjects had a significantly lower body weight (p=0.004) and borderline significantly lower BMI (p=0.050).

**Table 3:** Sex differences *donepezil*: Serum concentration and BMI of male and female participants, respectively, in the *donepezil* group.

Variables	male (n=21)	female (n=20)	p-value
Serum concentration donepezil [ng/ml]	34.75 ± 11.17 (18.75-56.65)	48.02 ± 16.83 (23.40-87.55)	0.004^b^
Body weight [kg]	74.4± 5.9 (60-83)	64.3 ± 7.8 (50-83)	<0.001^a^
BMI [kg/m²]	23.8 ± 2.4 (17.2-26.5)	23.8 ± 3.7 (18.6-34.5)	0.663^b^

ng, nanogram; ml, milliliter; kg, participants' weight in kilogram; BMI, body mass index; m^2^, square of body hight in meters. Where applicable: mean ± standard deviation (minimum - maximum). p-value calculated from ^a^t-test for normally distributed data and ^b^Mann-Whitney U test for not normally distributed data.

Characteristics and serum concentrations for the respective low and high concentration subgroup for donepezil and rivastigmine are shown in **Supplementary Table 6**. Serum concentrations differed significantly (p < 0.001) between the respective low concentration and high concentration subgroup. In the donepezil group only, there also was a statistically significant difference in the distribution of sex (p=0.008) with more males in the low concentration subgroup and more females in the high concentration subgroup.

#### Dose-Related Reference Range

According to the Consensus guidelines, the dose-related reference range for donepezil 10 mg daily is 44.20–63.80 ng/ml and for rivastigmine patch 9.5 mg it is 3.42–9.69 ng/ml (Hiemke et al., 2018). In the donepezil group, 63% (n=26) of participants had serum concentrations below that range, 32% (n=13) were within the range, and 5% (n=2) were above the range. In the rivastigmine group, 23% (n=6) were below the range, 54% (n=14) within, and 23% (n=6) above.

#### Genotype and Gene Dose

**Supplementary Table 7** shows tested alleles, the respective gene doses, and allele frequencies. For one subject, the analysis could not be completed. Gene dose distribution, type of metabolizer, and respective donepezil serum concentrations are shown in Supplementary

**Table 1**. Serum concentration in relation to gene dose and type of metabolizer is displayed in **Figure 1**.

**Figure 1:**
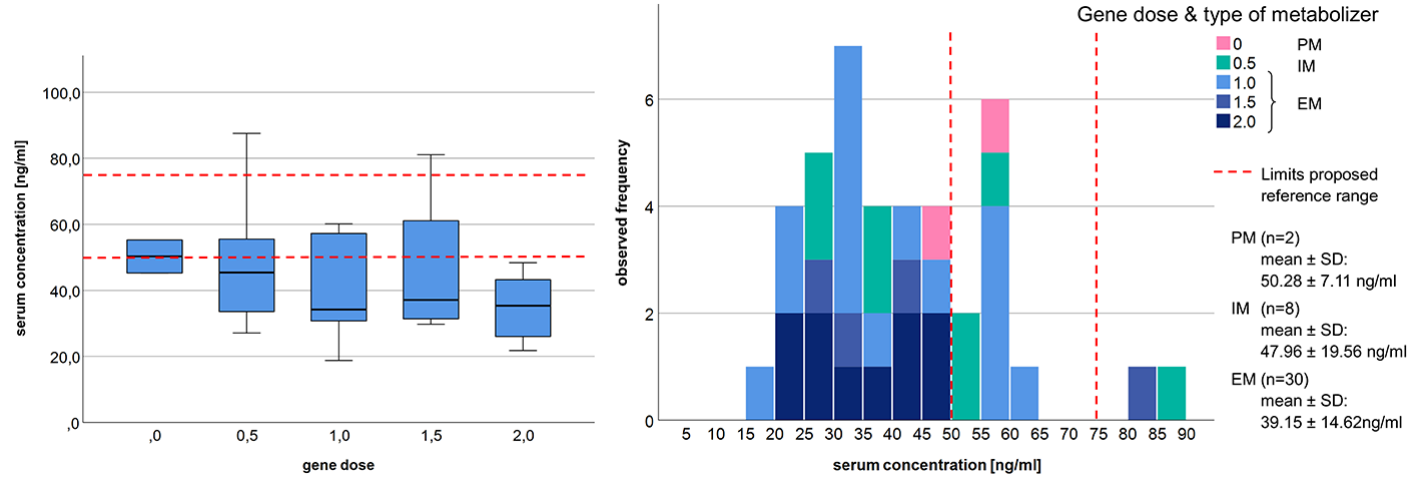
Distribution of donepezil serum concentration in regards to gene dose and the proposed therapeutic reference range (Hiemke et al., 2018). PM, poor metabolizer; IM, intermediate metabolizer; EM, extensive metabolizer; n, number of subjects; SD, standard deviation.

#### Correlation Between Donepezil Serum Concentration and CYP2D6 Gene Dose

**Figure 2:**
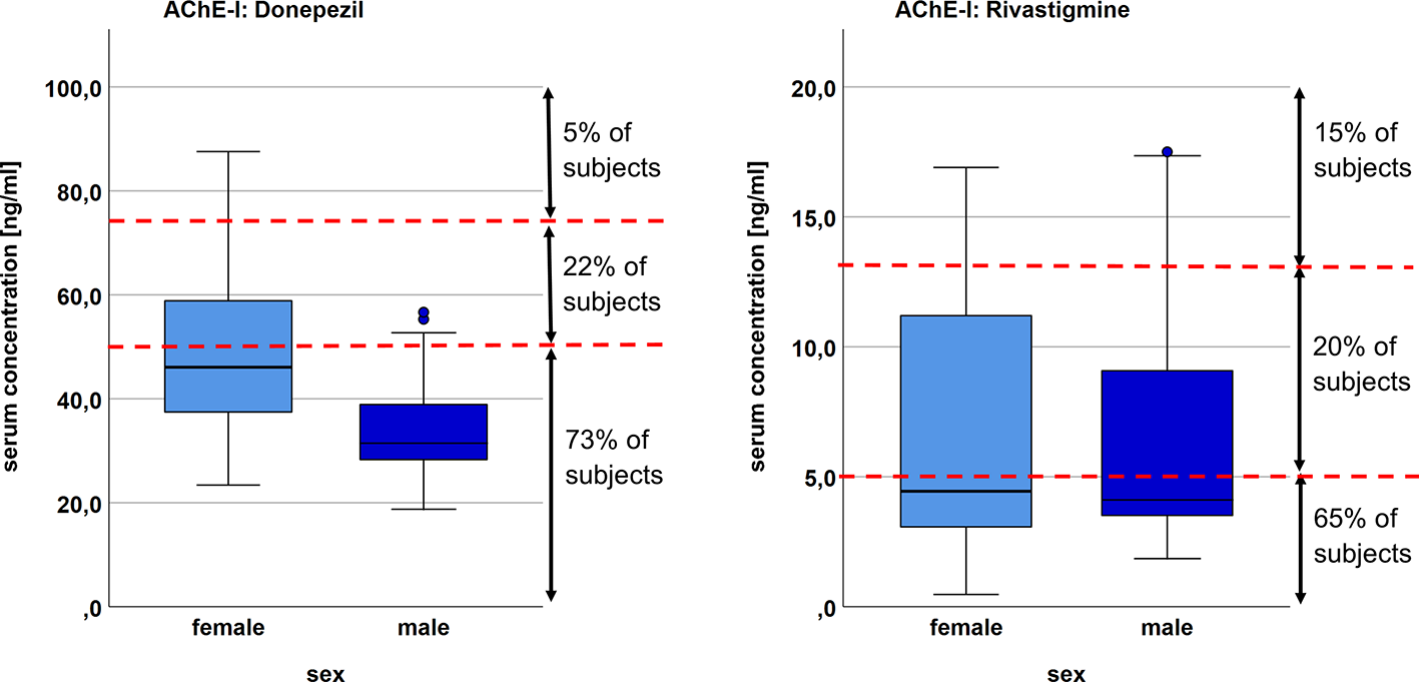
Distribution of serum concentration for female and male subjects in the donepezil and rivastigmine group, respectively. Red dotted line marks the limits of the proposed therapeutic reference range (donepezil 50–75 ng/ml; rivastigmine 5-13 ng/ml (Hiemke et al., 2018).

Gene dose (β=-0.375; p=0.046), sex (β=-0.742; p=0.001), and duration of donepezil treatment (β=0.341; p=0.030) were significant predictors of donepezil serum concentration and the applied regression model explained 35.1% of the variability of drug serum concentration (p=0.005) (**Table 4**). Sex explained most of the variability and differed significantly between the low and high concentration group (p=0.008). Serum concentrations in relation to sex and the therapeutic range are presented in **Figure 2**.

**Table 4:** Prediction of serum concentration of *donepezil: Multivariate* regression model; n = 37.

	Beta	p-value
Gene dose	-0.375	0.046
CYP2D6-Inhibitors	0.229	0.126
Age	0.195	0.186
Sex	-0.742	0.001
Duration of AChE-I treatment	0.341	0.030
Time since last dosing	-0.079	0.650
Body weight	0.171	0.343

AChE-I, acetylcholinesterase inhibitor.

corr. R²= 0.351, p = 0.005.

We found no indication for significant interactions, and the quality of the model did not improve after forcing interaction terms into the model. A table with the univariate regression analyses is shown in **Supplementary Table 8**.

To further explore variability of drug serum concentration, gene dose was replaced by metabolizer status in the multivariate regression model. This model yielded similar results with type of metabolizer and sex as significant predictors for donepezil concentration (**Supplementary Table 9**). The model was statistically significant (p=0.005) and explained 35.2% of the variability of serum drug concentration.

To rule out spurious correlation caused by differences in body weight between sexes, an additional linear regression analysis with BMI instead of body weight was calculated. The model was significant (p=0.005) and explained 34.9% of variability of serum drug concentration, with a similar pattern compared with the previous analysis. The effect of the gene dose just failed to reach significance (β=-0.354; p=0.061). Neither body weight nor BMI contributed significantly to the respective model (β=0.171 p=0.343 and β=0.127 p=0.372, respectively; **Table 4** and **Supplementary Table 10**).

#### Explorative Analyses of Influence Factors on Rivastigmine Drug Serum Concentration

As plausibility check, a regression analysis to explore possible predictors on serum concentration in the rivastigmine group was conducted including concomitant medication with CYP2D6 inhibitors, age, sex, duration of treatment with rivastigmine, time since last dosing, and body weight as independent variables. Neither the regression model (p=0,718; R²=-0,113) nor any of the variables were statistically significant (**Supplementary Table 11**). The models remained not significant after using BMI instead of body weight as independent variable (**Supplementary Table 12**).

### Correlations Between Changes in the Neuropsychological Test Results and Serum Concentrations of Donepezil and Rivastigmine

#### Changes in Neuropsychological Assessments Between Initial and Follow Up Assessment

There were no statistically significant differences between the donepezil and rivastigmine group regarding efficacy of AChE-I treatment based on the results of the CERAD-NAB subtest word list delayed recall (**Supplementary Table 4**).

#### Correlation Between Cognitive Changes and Drug Serum Concentration

Results of multivariate regression analyses for changes in the CERAD-NAB word list delayed recall subtest as dependent variable are shown in **Supplementary Table 13** for the donepezil and rivastigmine group.

The linear regression models to predict the word list delayed recall subtest were significant for both treatment groups. In both groups, the baseline results significantly predicted results at follow-up. Only in the rivastigmine group did drug serum concentration significantly contribute to the model (β = 0.472; p = 0.019) (**Supplementary Table 13**). When using “low/high serum concentration” as a factor, the goodness of fit for the model was similar for rivastigmine but no longer statistically significant (adjusted R²=0.294, p=0.098; **Supplementary Table 14**). For donepezil, the model got worse (adjusted R²=0.096, p=0.208) when using low/high serum concentration as a factor (**Supplementary Table 14**).

## Discussion

We investigated the correlation between donepezil serum concentration and the gene dose as a marker for CYP2D6 metabolism rate, and compared associations of serum concentrations and clinical efficacy of the hepatically metabolized donepezil and the renally excreted rivastigmine. Serum concentrations were below the recommended range in 73% of the subjects in the donepezil group and in 65% of the participants in the rivastigmine group. The gene dose significantly predicted donepezil serum concentration. Multiple linear regression models for changes in the subtest word list delayed recall were significant for both donepezil and rivastigmine, however, only serum concentration of rivastigmine significantly predicted changes in the test results. When using high or low rivastigmine serum concentration as a factor, the goodness of fit for the model with rivastigmine was similar, but the model was no longer significant.

### Drug Serum Concentrations and Gene Dose

Noteworthy, about two thirds of subjects in both groups had serum concentrations below the therapeutic range recommended by the AGPN Consensus Guidelines (Hiemke et al., 2018). As participants were either on a stable dosage of donepezil 10 mg or rivastigmine 9.5 mg, variability due to different dosages within one group can be ruled out.

More subjects in the donepezil group had lower than expected drug serum concentrations (73 vs. 65% in the rivastigmine group) and all but one of the subjects with a gene dose >1 fell in that category (compare **Figure 1**). Vice versa, being a poor metabolizer with a gene dose of 0 or 0.5 did not necessitate in a serum concentration within or above the recommended range. While in our study the gene dose, respectively type of metabolizer, significantly predicted donepezil serum concentration, previous studies investigating correlations between CYP2D6 mutations and donepezil concentration showed inconsistent results (Varsaldi et al., 2006; Seripa et al., 2011; Lu et al., 2014; Sonali et al., 2014; Miranda et al., 2017).

As 65% of the subjects treated with rivastigmine had low drug serum concentrations as well, we assumed that other factors than gene dose, such as sex or adherence, might contribute to the finding of lower than expected drug serum concentrations in this many subjects.

### Donepezil and Rivastigmine Serum Concentration in Context of the Recommended Therapeutic Range

Concerning donepezil, one reason might be that the current guideline increased the recommended lower limit from 30 to 50 ng/ml (Hiemke et al., 2011; Hiemke et al., 2018). Even adhering to the previous guideline, 25% of our subjects would have had serum concentration below the formerly recommended lower limit. Establishing a therapeutic range for each drug is not mandatory for pharmaceutical companies, so guidelines are subject to constant change and adaption, based on the availability of data. Within its therapeutic range, a drug should have a definite therapeutic effect while being tolerable and not harmful.

The Consensus Guidelines are based on published data about the respective drugs, were obtained from drug concentrations at therapeutically effective doses and related to clinical effects, and cut-off values were identified by receiver operating characteristic (ROC) analysis when possible (Hiemke et al., 2018).

Hefner et al. (2015) investigated in over 100 patients if donepezil serum concentration was associated with clinical improvement measured on the clinical global impression rating scale (CGI). They provided ROC analyses suggesting a serum concentration of at least 50 ng/ml was necessary for a good clinical response. Subjects that “very much improved” on the CGI actually had a mean serum concentration of 60 ng/ml. As Koeber et al. (2012) already suggested and as can be seen in our sample, a dosage higher than 10 mg might be necessary to achieve such serum concentrations of donepezil. We did not find comparable data on rivastigmine.

Although it has not specifically been investigated for AChE-I, most plasma and serum samples for measuring antidepressants or antipsychotics can be stored at 4°C in the dark for 24 h without affecting measuring of the respective drugs (Heller et al., 2004). Even if donepezil or rivastigmine were unstable at room temperature, it is unlikely that this would be the cause for the low serum concentrations we measured. In our study, blood samples were immediately stored at 4°C and centrifuged within 60 min of collection. Following centrifugation serum samples were stored at -20°C.

80% of subjects in our study had rivastigmine serum concentrations outside the recommended therapeutic range which is between 5–13 ng/ml (65% below, 15% above that range). We were surprised to not see a statistically significant association between rivastigmine serum concentration and body weight as this is described in the drug label information (FDA, 2012). Neither did we find another factor predicting serum concentration.

That an estimated half of medication in chronic diseases is not taken as prescribed might have contributed to overall low drug serum concentrations in this study (Zullig et al., 2013). Therefore we additionally looked at the dose-related reference range (Hiemke et al., 2018). In the rivastigmine group 54% of subjects were within recommended limits and each 23% above or below that range. Only 32% of participants in the donepezil group had serum concentrations within the dose-related reference range, 63% were below, and 5% above. Beside genetic abnormalities or drug interactions, low drug adherence is a likely cause for this finding. Noteworthy in this context seems the fact, that subjects treated with oral donepezil were 2.7 times as likely to have serum concentrations beneath the dose-related reference range as those treated with a transdermal rivastigmine patch (63 vs. 23%).

Another possible factor resulting in relatively more subjects with a sub-therapeutic concentration of donepezil might be found in the substrates of the respective medication. While donepezil causes a rapidly reversible inhibition of AChE, rivastigmine pseud-irreversibly inhibits both, AChE and BChE. When analyzing serum, a large proportion of AChE that is bound to the surface of RBC will have been removed from the sample, as will have the AChE-I that is bound to the enzyme. BChE as a soluble enzyme, on the other hand, will remain in serum and plasma. However, as rivastigmine binds quasi irreversibly to its target enzymes and is degraded during its interaction with the enzyme, measuring free rivastigmine in serum means that its binding sites are saturated. Measuring unchanged donepezil and rivastigmine-metabolites in whole blood might yield more precise results when investigating drug concentrations in terms of bioavailability. While a large number of studies investigated serum and plasma concentrations like we did and available data in the drug label information and guidelines report plasma or serum concentrations, some authors directly measured the activity and protein levels for AChE and BChE (Darreh-Shori et al., 2006; Nordberg et al., 2009; Darreh-Shori and Soininen, 2010; Darreh-Shori et al., 2014). Important findings from these studies are that donepezil, as a reversible, noncarbamylating AChE-I, more than doubles AChE protein expression and increases AChE activity in both, RBC and cerebro spinal fluid (CSF), and favors AChE-driven Aβ aggregation (Darreh-Shori et al., 2006; Nordberg et al., 2009; Darreh-Shori and Soininen, 2010; Darreh-Shori et al., 2014). This raises the question if increasing the dosage of donepezil would accelerate the progression of Alzheimer's pathology? Interestingly, Darreh-Shori et al. observed that adding low-dose phenserine, a carbamylating AChE, to donepezil treatment, the higher expression of AChE was counteracted (Darreh-Shori et al., 2014). Darreh-Shori et al. also found that CSF donepezil concentrations were higher after 24 months than after 12 month of treatment and that the inhibition of CSF AChE was 20% higher than RBC AChE inhibition, even though donepezil concentrations were almost 10 times lower in CNS as compared to plasma (Darreh-Shori et al., 2006). In contrast, rivastigmine, a pseudo-irreversible, carbamylating AChE-I and BChE-I, was associated with a decreased activity and decreased protein levels for both AChE and BChE (Nordberg et al., 2009; Darreh-Shori and Soininen, 2010).

### Drug Serum Concentration and Clinical Efficacy

#### Cognitive Symptoms

Rivastigmine serum concentration was the only significant predictor of changes in the subtest wordlist delayed recall. When using low/high serum concentration as a factor, regression models for both groups were no longer significant. However, roughly two thirds of study participants had drug serum concentrations below the recommended therapeutic range. As a result, when looking at the third of subjects with the highest serum concentrations in the donepezil group, some already had serum concentrations below the recommended range. In the rivastigmine group, the “high serum concentration” group comprised of all participants within or above the recommended reference range.

As a dose dependent, respectively blood concentration dependent effect has been suggested for both, donepezil and rivastigmine, low serum concentrations in our sample might explain lack of seen drug efficacy (Rogers et al., 1998; Chou et al., 2012; Yang et al., 2013; Hefner et al., 2015; Chen et al., 2017). In the US and some Asian countries, a 23 mg dosage of donepezil has been approved by regulation authorities (FDA, 2015) and although there were more adverse drug reactions, particularly of the gastrointestinal system, participants treated with 23 mg donepezil daily also showed significantly more improvement in cognition as compared to patients receiving a dosage of donepezil 10 mg (Farlow et al., 2010).

We looked at changes of cognitive symptoms between baseline and follow-up. As concomitant medication in subjects was stable between both assessments, we did not adjust test results for potential anticholinergic side effects of concomitant medication. Furthermore, the anticholinergic burden (ACB) score was low in our cohort (mean 0.42 ± 0.72) (Kiesel et al., 2018). Only one subject had an ACB score >2, a value above which a switch to alternative drugs is recommended (Boustani et al., 2008). Besides, the ACB scores did not significantly differ between groups (p = 0.487).

#### BPSD

A dose dependent treatment response has also been shown for BPSD in DLB. In patients with worsening of BPSD under treatment with 5 mg donepezil, Manabe et al. found that increasing the dosage to 10 mg led to statistically significant improvements on the Neuropsychiatric Inventory (NPI) (Manabe et al., 2016). Tan et al. also saw a dose dependent effect of donepezil on behavioral symptoms that was significant for 10 mg daily, but not for 5 mg (Tan et al., 2014). Beside Alzheimer's dementia, AChE-I are effective in dementia due to other neurodegenerative diseases. A Cochrane review found evidence of a positive impact of AChE-I for DLB, PDD, and cognitive impairment in PD (Rolinski et al., 2012). Beside cognitive functions, behavioral symptoms improved under treatment with AChE-I as well (Rolinski et al., 2012). Jin and Liu looked at the efficacy and safety of different drugs used to treat BPSD (Jin and Liu, 2019). They also found a positive effect for AChE-I. However, they also saw an increase in the risk of adverse events such as nausea and vomiting. Additionally, there might be sex-specific effects. On one hand, Matsuzono et al. (2015) described significantly improved scores on the Abe's Behavior and Psychological Symptom of Dementia Score (ABS) for female patients after three months of treatment with rivastigmine, but not for male patients. ABS for male patients, on the other hand, were stabilized under treatment with galantamine, while females deteriorated.

### Limitations

Sample size and variation of the time from baseline to follow-up appointments are the biggest limitations of this study. Due to the relatively small sample size, especially when further dividing treatment groups into a low and high serum concentration subgroup, weak correlations and small effects might not have been detected. Concerning time to follow-up, routine follow-up appointments were supposed to take place 3–6 months after the initial visit. However, there was much variability in the actual time span to follow-up appointments. As duration of treatment had a statistically significant influence on donepezil serum concentration in our regression model, a longer treatment period might have resulted in higher serum concentrations and possibly higher efficacy. Although according to drug label information, steady state is reached after about 15 days of treatment with donepezil (FDA, 2015), Miranda et al. also described increasing serum concentrations over a study period of one year (Miranda et al., 2017) and Darreh-Shori et al. reported a further increase of donepezil concentration in CSF after 24 months of treatment.

Some cited studies measured drug concentrations in serum, some in plasma. We decided to use serum, as this constitutes the standard for measuring drug concentrations in Germany. As serum equals plasma without fibrinogen, measurements should be identical, especially when using Liquid Chromatography Mass Spectrometry. We are not aware of any studies comparing donepezil or rivastigmine concentrations in plasma to that in serum and the current Guidelines for Therapeutic Drug Monitoring in Neuropsychopharmacology considers both, serum and plasma, as equal (Hiemke et al., 2018).

We aimed to measure drug serum concentration in the trough state as close to the next scheduled dosing as possible. In some cases, however, this was not possible. While the effect on the serum concentrations of donepezil with a half-life of 70 h (FDA, 2015) might have been small, holding the next dose of rivastigmine with a half-life of 3.4 h after patch administration (FDA, 2012) for up to 4 h might have had a bigger effect on rivastigmine concentrations.

Another limitation is that we did not measure mutations in the BChE gene. As BChE wild-type carriers have been reported to show greater response to rivastigmine than donepezil (Blesa et al., 2006) and as some variants, such as BChE-K, cause reduced AChE activity (O'Brien et al., 2003) and thereby reduced degradation of rivastigmine, this might have affected measures of efficacy as well as rivastigmine serum concentration.

Assessing efficacy on cognition in a steadily progressing disease with day to day fluctuation poses a challenge. Even if no improvement is seen, slower worsening has to be considered as treatment effect. In small samples and without a placebo group, however, the effect might be easily missed. While the CERAD-NAB is a well-established test for Alzheimer's disease, it does not assess activities of daily living or BPSD. It would have been of use if additional tests for the ability of daily living and BPSD would have been administered for this study. Improvement in these fields may have occurred even in the absence of improvement on the CERAD-NAB sub-score for word list delayed recall.

Last but not least, information about treatment adherence was obtained from the caregivers and may have been flawed as indicated by low serum concentrations compared to the dose-related reference ranges. Questions regarding drug adherence may have been answered to please the treating physician.

### Conclusions

Since there are signs that a substantial number of subjects treated with AChE-I have drug serum concentrations below the recommended reference range, therapeutic drug monitoring might improve the efficacy of AChE-I when treating cognitive and behavioral symptoms in dementia. This might be true not only for ADD, but also for PDD, DBL, and possibly vascular dementia. Studies on different forms of dementia investigating drug blood concentration while assessing not only cognitive tests, but also scales for activity of daily living, BPSD and caregiver burden would be of value. As donepezil and rivastigmine have fundamentally different effects on AChE concerning protein concentration and activity, measuring plasma or serum concentrations might not be quite as meaningful when assessing the effect of AChE-I. There also might be room for improvement on medication adherence. Thorough education of patients and caregivers or the involvement of home nursing might be of benefit when seeking top treatment result. In addition, drug adherence seems to be better in a transdermal application of the AChE-I.

## Data Availability Statement

The datasets used and/or analysed during the current study are available from the corresponding author on reasonable request. However, due to the nature of pseudonymized patient data, a material transfer agreement is required to meet ethical standards and data privacy laws of Germany.

## Ethics Statement

The studies involving human participants were reviewed and approved by Faculty of Medicine of the Technical University of Munich, Munich (reference number 673/02). The patients/participants provided their written informed consent to participate in this study.

## Author Contributions

MO: Design of the study, analysis and interpretation of data, drafting the manuscript. MS: Acquisition, analysis and interpretation of data, drafting the manuscript. HS: Analysis and interpretation of data, drafting the manuscript, revising the manuscript for intellectual content. CS: Analysis and interpretation of data, revising the manuscript for intellectual content. KB: Acquisition and analysis of the data, revising the manuscript for intellectual content. CM: Acquisition and analysis of data, revising the manuscript for intellectual content. BD: Collecting data, revising the manuscript for intellectual content. OG: Collecting data, revising the manuscript for intellectual content. JD-S: Acquisition and interpretation of data, and revising the manuscript for intellectual content. HF: Interpretation of data and revising the manuscript for intellectual content. WS: Conceptualization of the study, interpretation of data, and revising work for intellectual content. TG: Conceptualization of the study, analysis and interpretation of data, drafting and revising the manuscript for intellectual content. All authors approved of the final version of the manuscript and agreed to be accountable for all aspects of the work.

## Funding

This work was supported by the German Research Foundation (DFG) and the Technical University of Munich (TUM) in the framework of the Open Access Publishing Program. CYP2D6 genotyping was supported by an unrestricted research grant by Novartis. Novartis was neither involved in data collection, measurements or statistical analyses nor in the interpretation of the data.

## Conflict of Interest

All authors reported no disclosures with regards to the submitted work. Outside the submitted work, TG reported having received consulting fees from Actelion, Biogen, Eli Lilly, Iqvia/Quintiles; MSD; Novartis, Roche Pharma, lecture fees from Biogen, Lilly, Parexel, Roche Pharma, and grants to his institution from Actelion and PreDemTech. OG reported having received a consulting fee from Eli Lilly and grants to his institution from Actelion. JD-S reported having received lecture fees from Novartis. WS reported having received consulting fees, support or lecture fees from Roche Diagnostics, Abbott, Siemens, Microgenics, Thermo Fisher.

The remaining authors declare that the research was conducted in the absence of any commercial or financial relationships that could be construed as a potential conflict of interest.
