# Supplementary material for: Serum Concentrations of Cholinesterase Inhibitors in Patients With Alzheimer’s Dementia Are Frequently Below the Recommended Levels
Source: Front Pharmacol. 2020 May 21;11:691. doi: 10.3389/fphar.2020.00691 (PMC7253642; doi:10.3389/fphar.2020.00691)
Supplement: Supplementary file 1 [file DataSheet_1.pdf]

Supplementary Materials

Methods

Blood sample collection

Whole blood venous samples were collected with and without anticoagulant (ethylenediaminetetracetic acid (EDTA)). EDTA samples were immediately stored at -30 degree Celsius (°C) until deoxyribonucleic acid (DNA) extraction. Serum samples were immediately stored at 4°C and centrifuged for 9 minutes at 2300 g and 4°C within 60 minutes after collection. Serum was removed and stored at -20°C. To avoid further freeze and thaw cycles serum samples were thawed once and stored in aliquots (1.5 ml reaction tubes).

Liquid chromatography-mass spectrometry methods

The method was optimized starting from the method described by Noetzli et al. 2012 [1]. Validation was carried out according to the guideline C62-A for Liquid Chromatography-Mass Spectrometry Methods [2].
A triple quadrupole mass spectrometer (QTRAP 5500, SCIEX, USA) with a high-performance liquid chromatography (HPLC) system (1260, G1367E; Agilent Technologies Deutschland GmbH, Germany) was used to analyze the samples in SRM mode (selected reaction monitoring mode). For the quantification of donepezil mass transition 381->91.1(Da) was selected and 383.5 -> 91.0 (Da) for IS (donepezil $^{13}C_3$). Transiton 251.1->58.0 (Da) combined with 257.1 ->206.1 (Da) (rivastigmine D_6) was used for rivastigmine quantification. For data analysis Analyst 1.6 (SCIEX Pte.Ltd, Singapore) was applied.
A seven point calibration curve was prepared (0.5 ng/ml to 33 ng/ml for rivastigmine and 0.5 ng/ml to 66 ng/ml for donepezil) using drug free serum. In addition, three independent quality control samples with concentrations covering the calibration ranges were prepared.
For sample preparation 50 µl serum was mixed with 200 µl precipitation agent for 15 seconds at 1000 rpm. The precipitation agent (acetonitrile/methanol 30/70(v/v)) contained internal standards (IS): 10 ng/ml rivastigmine D_6 (Toronto Research Chemicals (TRC), Toronto, Ontario) and donepezil $^{13}C_3$ 50 ng/ml (TRC, Toronto Ontario). The mixed sample was centrifuged at 1000 x g for 10 minutes. Either 10 µl or 50 µl of the supernatant were injected into the LC-MS system to analyze donepezil and rivastigmine, respectively. Chromatographic separation was carried out using a gradient on a reversed phase C_{18} column (Onyx Monolithic C18, 100 x 3.0 mm, Phenomenex Germany, Germany) at 45°C. Mobile phase A was of H_2O, mobile phase B was methanol, each buffered with 0.1 % acetic acid (v/v) and 12.5 mM ammonium acetate. All solvents were LC-MS grade. Total run time was 7 minutes.
Patient samples above the highest calibration concentration were diluted with drug free serum. All samples were measured at least in duplicate and mean was used for further analyses.

Assessment of CYP2D6 polymorphisms and gene dose assignment

DNA was isolated by standard procedures from 300 µl EDTA anticoagulated blood according to the manufacturer's instructions (Wizard Genomic DNA Purification Kit, Promega). The isolated DNA of the CYP2D6 gene was immediately preamplified according to local SOPs. Considering the prevalence of CYP2D6 polymorphisms in Caucasians, the following alleles were assessed: *1 (wildtype), *3, *4, *5, *6 (no enzyme activity), *9, *10, *41 (reduced enzyme activity), and duplications of *1 (increased enzyme activity). Genotyping for all polymorphisms of interest was performed according to methods previously described using real-time quantitative polymerase chain reaction (PCR) [3-5] on a LightCycler 2.0 (Roche, Mannheim) with LightCycler Carousel Centrifuge (Roche, Mannheim). Following previously published methods [6-8] allele specific PCR was performed using GeneAmp PCR Systems 9700 (PE Applied Biosystems, Weiterstadt) for CYP2D6*3, CYP2D6*4, CYP2D6*5,

CYP2D6*6 and CYP2D6*10. Supplementary table 1 shows assessed polymorphisms and used methods of assessment.
Defective alleles were assigned a gene dose of 0, alleles resulting in a reduced enzyme activity were assigned a gene dose of 0.5, wild type allele *1 a gene dose of 1, and duplications of a wild type allele a gene dose of at least 2 (supplementary table 2).

References:

1 Noetzli M, Ansermot N, Dobrinas M, Eap CB. Simultaneous determination of antidementia drugs in human plasma: procedure transfer from HPLC-MS to UPLC-MS/MS. J Pharm Biomed Anal. 2012;64-65:16–25. doi:10.1016/j.jpba.2012.02.008.
2 Clarke W, Molinaro RJ. Liquid chromatography-mass spectrometry methods: Approved guideline. Wayne, Penn.: Clinical and Laboratory Standards Institute; 2014.
3 Raimundo S, Toscano C, Klein K, Fischer J, Griese E-U, Eichelbaum M, et al. A novel intronic mutation, 2988GA, with high predictivity for impaired function of cytochrome P450 2D6 in white subjects. Clin Pharmacol Ther. 2004;76:128–38. doi:10.1016/j.clpt.2004.04.009.
4 Müller B, Zöpf K, Bachofer J, Steimer W. Optimized strategy for rapid cytochrome P450 2D6 genotyping by real-time long PCR. Clin Chem. 2003;49:1624–31.
5 Stamer UM, Bayerer B, Wolf S, Hoeft A, Stüber F. Rapid and reliable method for cytochrome P450 2D6 genotyping. Clin Chem. 2002;48:1412–7.
6 Ji L, Pan S, Marti-Jaun J, Hänseler E, Rentsch K, Hersberger M. Single-step assays to analyze CYP2D6 gene polymorphisms in Asians: Allele frequencies and a novel *14B allele in mainland Chinese. Clin Chem. 2002;48:983–8.
7 Sachse C, Brockmöller J, Bauer S, Roots I. Cytochrome P450 2D6 variants in a Caucasian population: Allele frequencies and phenotypic consequences. Am J Hum Genet. 1997;60:284–95.
8 Løvlie R, Daly AK, Molven A, Idle JR, Steen VM. Ultrarapid metabolizers of debrisoquine: Characterization and PCR-based detection of alleles with duplication of the CYP2D6 gene. FEBS Lett. 1996;392:30–4.

Supplementary table 1

Semiquantitative gene dose	Mutation of alleles	Type of metabolizer	Number of participants	Donepezil serum concentration [ng/ml]
0	2 deficient alleles	PM	2	50.3 ± 7.1 (45.3-55.3)
0.5	1 deficient allele + 1 reduced allele	IM	8	48.0 ± 19.55 (27.1-87.6)
1	2 reduced alleles/ 1 deficient allele + 1 wt	EM	16	39.9 ± 14.7 (18.8-60.2)
1.5	1 wt + 1 reduced allele	EM	4	46.2 ± 23.7 (29.8-81.1)
2	2 x wt	EM	10	35.1 ± 10.1 (21.8-48.4)
≥ 3	duplication	UM	0	-

Supplementary table 1 Gene dose, allele mutations and type of metabolizer: wt: wild type; PM: poor metabolizer; IM: intermediate metabolizer; EM: extensive metabolizer; UM: ultra-rapid metabolizer

Supplementary table 2

Polymorphism	SOP Thermocycler	SOP LightCycler
Deletion (*5)/Duplikation	C2D6 Del-Dup-P PCR Del-Dup	C2D6 Del-Dup LC
*3	C2D6 multiplex-P PCR multiplex	C2D6 multiplexB-S probe multiplexB
*4, *6	C2D6 multiplex-P PCR multiplex	C2D6 multiplexA-S probe multiplexA
*9	-	C2D6 9-S probe-9
*10	C2D6 10-P PCR-10	C2D6 10-S probe-10
*41	-	C2D6 41-S probe-41

Legend supplementary table 2 Overview of polymorphisms and applied measuring methods: SOP: standard operating procedure; C2D6: Cytochrome P450 isoenzyme 2D6; Del: deletion; Dup: duplication; PCR: Polymerase Chain Reaction

Supplementary table 3

Allele	*1	*3	*4	*5 (Del)	*6	*9	*10	*41	Duplication
Single gene dose	1	0	0	0	0	0.5	0.5	0.5	>2

Legend supplementary table 3 Single alleles and corresponding gene dose: Del: deletion.

Supplementary table 4

	n=	Donepezil	n=	Rivastigmine	p-value
baseline: word list delayed recall	41	2.0 ± 1.8 (0 to 7)	26	2.4 ± 2.0 (0 to 7)	0.431
Follow-up: word list delayed recall	41	0.4 ± 1.5 (-2 to 4)	25	0.2 ± 1.4 (-2 to 3)	0.384

Supplementary table 13: word list delayed recall at baseline and at follow-up; n: number of valid test results; where applicable: mean ± standard deviation (minimum – maximum); p-value calculated from Mann-Whitney U test

Supplementary table 5

**Variables**	**male** (n=16)	**female** (n=10)	**p-value**
Serum concentration rivastigmine [ng/ml]	6.63 ± 5.28 (1.85-17.50)	6.39 ± 5.19 (0.47-16.90)	1.000[b]
Body weight [kg]	74.1 ± 12.1 (50-90)	58.4 ± 10.7 (47-80)	0.004[a]
BMI [kg/m²]	24.6 ± 2.59 (19.5-27.8)	21.6 ± 4.5 (16.5-30.9)	0.050[a]

Legend supplementary table 5 Sex differences *rivastigmine*: Serum concentration and BMI of male and female participants, respectively, in the *rivastigmine* group.
ng: nanogram; ml: milliliter; mg: milligram; kg: participants' weight in kilogram; BMI: body mass index. Where applicable: mean ± standard deviation (minimum - maximum). p-values calculated from [a]: t-test for normally distributed data; [b]: Mann-Whitney U test for not normally distributed data

Supplementary table 6

**Variables**	**Donepezil low concentration**	**Donepezil high concentration**	**p-value**	**Rivastigmine low concentration**	**Rivastigmine high concentration**	**p-value**
Numbers	14	14		9	9	-
Sex: male:female [n]	11 : 3 (79% : 21%)	4 : 10 (29% : 71%)	**0.008** [a]	6 : 3 (67% : 33%)	5 : 4 (56% : 44%)	0.629 [a]
Age [years]	69.9 ± 10.2 (53-88)	74.3 ± 9.4 (54-85)	0.244 [b]	73.6 ± 4.6 (69-82)	70.6 ± 7.7 (53-78)	0.329 [b]
Time baseline to follow up [days]	226.7 ± 87.7 (96-441)	214.2 ± 63.9 (131-325)	0.839 [c]	248.6 ± 99.7 (122-442)	215.7 ± 40.7 (164-259)	0.666 [c]
Duration from start of AChE-I treatment to follow up [months]	5.9 ± 3.3 (3-13)	5.6 ± 2.0 (3-10)	0.839 [c]	6.0 ± 2.6 (4-12)	5.4 ± 1.7 (3-8)	0.931 [c]
Participants taking CYP2D6 inhibitors [n]	2 (14%) (2x Citalopram)	4 (29%) (3x Citalopram; 1x Carvedilol)	0.357 [a]	n.a.	n.a.	-
Participants taking CYP2D6 inducers [n]	-	-	-	n.a.	n.a.	-
Weight [kg]	70.2 ± 9.1 (50-83) n=13	68.6 ± 9.1 (53-83) n=13	0.650 [c]	74.9 ± 10.8 (59-89) n=8	63.6 ± 15.9 (47-84)	0.139 [c]
BMI [kg/m²]	22.9 ± 2.9 (17.2-26.2) n=13	24.1 ± 3.7 (19.7-34.5) n=13	0.650 [c]	25.7 ± 2.7 (22.6-30.9) n=8	22.1 ± 4.3 (16.5-27.7)	0.074 [c]
Gene dose	1.3 ± 0.6 (0.5-2)	1.0 ± 0.6 (0-2)	0.128 [c]	n.a.	n.a.	-
Serum concentration [ng/ml]	26.76 ± 4.14 (18.75-31.45)	58.82 ± 11.79 (46.90-87.55)	**<0.001** [c]	2.67 ± 1.01 (0.47.3.62)	12.50 ± 4.46 (0.47.3.62)	**<0.001** [c]
Variance of serum concentration	17.16	138.93	**<0.001** [d]	1.01	19.10	**<0.001** [d]
Variation coefficient of serum concentration measurement	5.47 ± 3.01 (1.46-10.32)	5.21 ± 2.26 (0.77-9.09)	0.910 [c]	5.88 ± 4.62 (2.54-17.40)	5.28 ± 3.82 (1.00-10.99)	0.863 [c]
Time since last dosing [h]	21.0 ± 2.6 (14-25)	22.6 ± 1.9 (19-26)	0.050 [c]	23.6 ± 4.1 (15-28)	24.4 ± 1.6 (22-27)	0.931 [c]
Wordlist recall at baseline	2.3 ± 1.7 (0-5)	1.4 ± 1.6 (0-5)	0.164 [c]	2.1 ± 2.7 (0-7)	3.1 ± 2.1 (0-6)	0.297 [c]
Changes wordlist recall	0.1 ± 2.0 (-2 to 4)	0.4 ± 1.6 (-1 to 2)	0.482 [c]	0 ± 1.6 (-2 to 2)	0.8 ± 1.3 (0-3)	0.161 [c]

Legend supplementary table 6 Characteristics of participants in the low and high serum concentration group for donepezil and rivastigmine: where applicable: mean ± standard deviation (minimum - maximum); AChE-I: acetylcholinesterase inhibitor; kg: participants' weight in kilogram; BMI: body mass index in kilogram per square meter; p-values calculated from [a]: Chi-squared test, [b]: t-test or [c]:Mann-Whitney-U-test; [d]: F-test.

Supplementary table 7

Allele	*1	*3	*4	*5 (Del)	*6	*9	*10	*41	Duplication *1
Single gene dose	1	0	0	0	0	0.5	0.5	0.5	2
Number	40	1	23	3	1	1	2	9	0
Allele frequency	0.500	0.013	0.288	0.038	0.013	0.013	0.025	0.113	-

Legend Supplementary table 7 CYP2D6 polymorphism: Overview of tested alleles, corresponding gene dose, absolute number and frequency of alleles. Del: Deletion

Supplementary table 8

	Gene dose	CYP 2D6-Inhibitors	Age	Sex	Duration AChE-I intake	Time since last dosing	Body weight
corr. R^2	0.047	0.044	0.005	0.165	-0.025	0.054	-0.011
Beta	-0.267	0.261	0.172	-0.432	-0.017	0.279	-0.132
p-value	0.096	0.099	0.282	0.005	0.915	0.077	0.435

Legend supplementary table 8 Prediction of serum concentration of *donepezil univariate* regression analyses: AChE-I: acetylcholinesterase inhibitor.

Supplementary table 9

	Beta	p-value
Type of metabolizer	-0.314	0.045
CYP2D6-Inhibitors	0.275	0.060
Age	0.180	0.216
Sex	-0.734	0.001
Duration of AChE-I treatment	0.286	0.058
Time since last dosing	0.009	0.954
Body weight	0.200	0.269

Legend supplementary table 9 Prediction of serum concentration of *donepezil using type of metabolizer instead of gene dose. Multivariate* regression model. AChE-I: acetylcholinesterase inhibitor. Corr R^2=0.352, p=0.005

Supplementary table 10

	Beta	p-value
Gene dose	-0.354	0.061
CYP2D6-Inhibitors	0.240	0.112
Age	0.195	0.187
Sex	-0.630	<0.001
Duration of AChE-I treatment	0.327	0.035
Time since last dosing	-0.065	0.710
BMI	0.127	0.372

Legend supplementary table 10: Prediction of serum concentration of *donepezil: Multivariate* regression model *including body mass index (BMI) instead of weight*. AChE-I: acetylcholinesterase inhibitor. Corr R^2=0.349, p=0.005

Supplementary table 11

	Beta	p-value
CYP2D6-Inhibitors	0.081	0.773
Age	-0.098	0.684
Sex	0.138	0.635
Duration AChE-I treatment	0.096	0.753
Time since last dosing	0.432	0.104
Body weight	-0.276	0.351

Legend supplementary table 11 Prediction of serum concentration of *rivastigmine: Multivariate* regression model including body weight; AChE-I: acetylcholinesterase inhibitor. Corr. R^2=-0,113, p=0,718

Supplementary table 12

	Beta	p-value
CYP2D6-Inhibitors	0.136	0.625
Age	-0.083	0.722
Sex	0.125	0.614
Duration AChE-I treatment	0.050	0.869
Time since last dosing	0.438	0.087
BMI	-0.348	0.186

Legend supplementary table 12 Prediction of serum concentration of *rivastigmine: Multivariate* regression model including body mass index; AChE-I: acetylcholinesterase inhibitor; BMI body mass index. Corrected R^2=-0,055, p=0,583

Supplementary table 13

		Serum concentration	Baseline test result	Time baseline to follow-up	Sex	Age	model corr. R^2	model p-value
Word list – delayed recall donepezil	Beta	0.094	-0.445	-0.131	0.160	-0.156	0.169	0.041
	p-value	0.571	0.008	0.385	0.342	0.307		
Word list – delayed recall rivastigmine	Beta	0.472	-0.622	0.281	0.244	-0.194	0.267	0.049
	p-value	0.019	0.006	0.155	0.199	0.297		

Legend supplementary table 14 Correlation between cognitive changes and AChE-I serum concentration: *multivariate* regression analyses between changes in word list delayed recall and *donepezil* respectively *rivastigmine* serum concentration, baseline test result, time from baseline to follow-up, sex, and age.

Supplementary table 14

		Low /high serum concen-tration	Baseline test result	Time baseline to follow-up	Sex	Age	model corr. R^2	model p-value
Word list – delayed recall donepezil	Beta	0.055	-0.447	-0.102	0.114	-0.171	0.096	0.208
	p-value	0.814	0.044	0.597	0.634	0.415		
Word list – delayed recall rivastigmine	Beta	0.542	-0.711	0.503	0.392	-0.211	0.294	0.098
	p-value	0.035	0.014	0.044	0.110	0.357		

Legend supplementary table 15 Correlation between cognitive changes and low / high AChE-I serum concentration: *multivariate* regression analyses between changes in cognitive tests and *donepezil* respectively *rivastigmine* low / high serum concentration, baseline test result, time from baseline to follow-up, sex, and age.

Supplementary figures

Supplementary figure 1

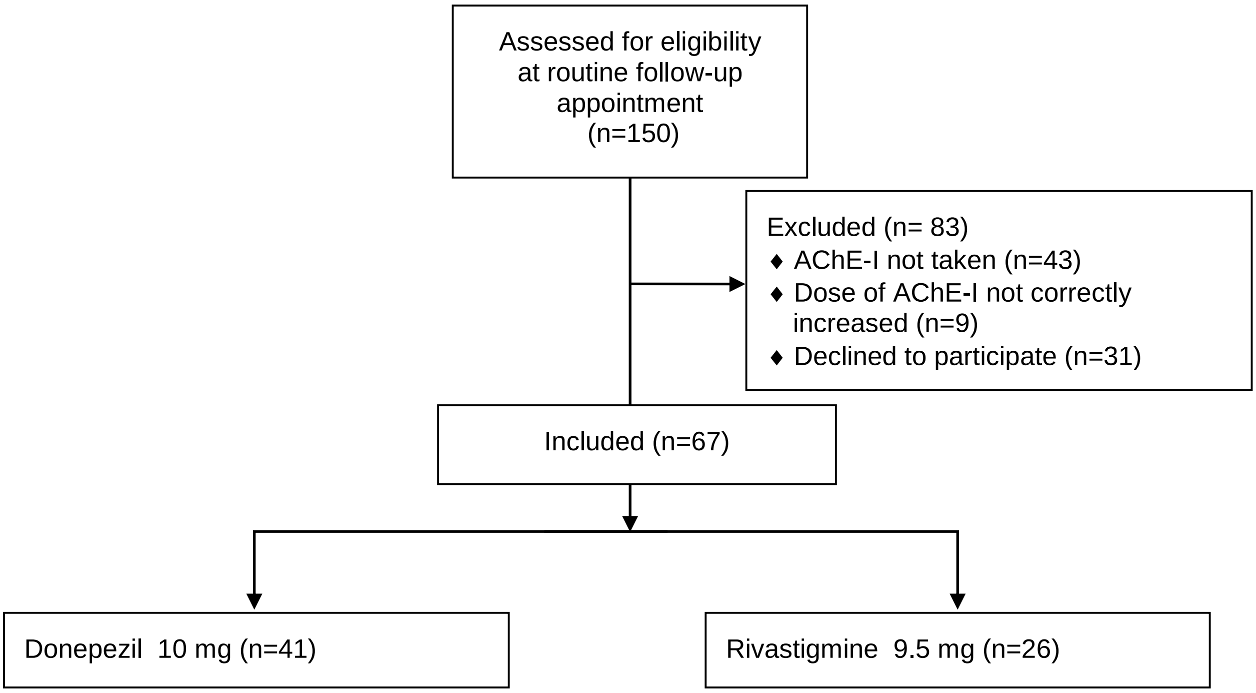

Legend to supplementary figure 1: Consort Flow Diagram depicting progress of participants
n: number of subjects; AChE-I: acetylcholinesterase inhibitor.
